# PPARα agonist relieves spinal cord injury in rats by activating Nrf2/HO-1 via the Raf-1/MEK/ERK pathway

**DOI:** 10.18632/aging.203699

**Published:** 2021-11-19

**Authors:** Haocong Zhang, Dulei Xiang, Xinwei Liu, Liangbi Xiang

**Affiliations:** 1 The Department of Orthopedics, General Hospital of Northern Theater Command of Chinese People’s Liberation Army, Shenyang, Liaoning, China; 2 Jinzhou Medical University Graduate Training Base of General Hospital of Northern Theater Command, Jinzhou, Liaoning, China

**Keywords:** PPARα agonist PEA, spinal cord injury, Nrf2/HO-1, Raf-1/MEK/ERK, TUNEL staining

## Abstract

Objective: To observe the inhibitory effects of the peroxisome proliferator-activated receptor alpha (PPARα) agonist palmitoylethanolamide (PEA) on inflammatory responses and oxidative stress injury in rats with spinal cord injury (SCI).

Methods: The SCI rat model was established using modified Allen's method and the changes in rats’ joint motion were observed by Basso, Beattie and Bresnahan locomotor rating scale (BBB scale) at 1, 3 and 7 days after modeling, HE Staining and Nissl Staining has been carried out to evaluate the pathological lesion of spinal cords in rats. Besides, Immunohistochemical (IHC) was performed to detect the reactive oxygen species (ROS), expression levels of acrylamide (ACR) and manganese superoxide dismutase (MnSOD) in rat spinal cords, and Western Blotting was applied to measure protein expression levels of nuclear factor-kappa B (NF-κB), B cell lymphoma-2 (Bcl-2), BCL-2 associated X (BAX), phosphoinositide 3-kinase (PI3K), protein kinase B (Akt), phosphorylated (p)-Akt, HO-1, Nrf2, trithorax-1 (TRX-1), Raf-1, MEK, ERK, p-MEK and p-ERK.

Results: The PPARα agonist PEA could alleviate SCI in rats, inhibit inflammatory responses, mitigate oxidative stress injury, reduce the apoptotic rate and promote SCI rats motor function recovery. In addition, the PPARα agonist PEA was able to activate the phosphorylation of MEK and ERK, stimulate Nrf-2 translocation into the nucleus and up-regulate the expressions of HO-1 and TRX-1.

Conclusion: PPARα agonist PEA can relieve SCI in rats by inhibiting inflammatory responses and oxidative stress, which may involve a mechanism associated with the activation of Nrf2/HO-1 via the Raf-1/MEK/ERK pathway.

## INTRODUCTION

Spinal cord injury (SCI) is a serious nerve injury caused by the destruction of nerve cells’ axons, which can lead to limb dysfunction below the injured segments. It can be classified into primary and secondary injuries [1]. The primary injury is caused by an external force and is irreversible, while the secondary injury refers to SCI that is further aggravated by a series of primary injury-initiated complex processes, and in some cases, the latter is severer than the former [2]. Following SCI, the inflammatory responses and oxidative stress injury are crucial events of the secondary injury; therefore, developing effective prevention and treatment measures, that address the issue at the source of pathogenesis, is of important clinical significance in improving SCI prognosis [3, 4].

In the nervous system neuroblasts, the role of the V-raf-1 murine leukemia viral oncogene homolog 1 (Raf-1)/mitogen-activated protein kinase (MEK)/extracellular signal-regulated kinase (ERK) signal transduction, in regulating genes expression, has already been proved [5]. Raf is inactive in resting cells and mainly localized in the cytoplasm. After activation by Ras, Raf accumulates on the cell membrane inner surface, where its conformation is altered and exposes its phosphorylation sites [6]. The phosphorylated Raf monomers form heterologous oligomers to achieve full activation, which specifically catalyzes.

MEK1 and MEK2 phosphorylation and activates ERK1/2 to translocate from the cytoplasm to the nucleus [7, 8], thereby participating in biological responses, such as cell proliferation and differentiation, maintenance of cell morphology, cell inflammatory responses, apoptosis and malignant transformation. These events are mediated by the transcriptional activation of ELK-1, interleukin-1 beta (IL-1β) and nuclear factor-kappa B (NF-κB) [9].

As a vital member of the peroxisome proliferators-activated receptor (PPAR) family, PPARα serves as an essential ligand-activated transcription factor that represses inflammatory responses by inhibiting pro-inflammatory cytokines’ products, adhesion molecules and extracellular matrix proteins [10, 11]. The anti-inflammatory mechanism of PPARα is primarily associated with the activation of the nuclear factor erythroid 2-related factor 2 (Nrf2) and the inhibition of NF-κB. The previous studies have identified that PPARα plays crucial role and is widely involved in the progression of SCI [12–14]. Importantly, the endogenous fatty acid palmitoylethanolamide (PEA), as a PPARα agonist, is one of the members of N-acyl-ethanolamines family [15]. The previous investigations have identified that the administration of PEA explicates multiple impacts on central nervous system (CNS), including protecting against 1-methyl-4-phenyl-1,2,3,6-tetrahydropyridine (MPTP)-related memory and learning impairment and neurotoxicity, regulating inflammatory pain, affecting pentobarbital-evoked hypnotic effect, inducing the neurotrophic substance initiation in SCI, and attenuating SCI-induced tissue injury and inflammation [15–22]. Despite the biological effect of PEA has been well reported, the molecular mechanisms underlying PEA-mediated SCI remain unclear.

Previous studies have demonstrated that Nrf2 is dissociated from the Nrf2/Kelch-like ECH-associated protein 1 (Keap1) complex after organisms’ stimulation by oxidative stress or exogenous substances, thereby triggering its nuclear translocation. There is a bidirectional regulatory relationship between PPARα and NF-κB. The activated PPARα can induce the rise in NF-κB inhibitor (IKB-α) expression and inhibit the translocation of NF-κB into the nucleus and its conjugation with DNA, thus directly repressing the expression of NF-κB-mediated inflammatory factors and shortening the process of inflammatory responses. Hence, it was conjectured in this study that PPARα may suppress SCI inflammation and alleviate oxidative stress injury through a mechanism involving the Raf-1/MEK/ERK signaling pathway [23, 24].

In this study, we were interested in the role and related mechanism of PPARα agonist PEA in the regulation of SCI. We identified the crucial effect of PEA in relieving SCI in rats by activating Nrf2/HO-1 via the Raf-1/MEK/ERK pathway.

## RESULTS

### PPARα agonist improved BBB score and relieved SCI in SCI rats

**Figure 1:**
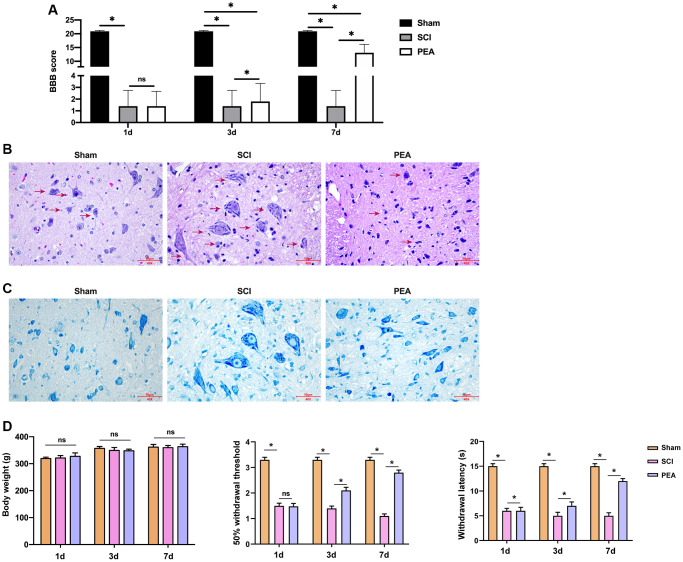
**PPARα agonist improved BBB score and relieved SCI in SCI rats.** (**A**–**D**) The rats were randomly divided into sham-operation group (Sham group), rat SCI model group (SCI group), SCI + peroxisome proliferator-activated receptor alpha (PPARα) agonist PEA group (PEA group). The SCI rat model was established using modified Allen's method. The rats in the PEA group were intraperitoneally injected with PEA (2 mg/kg). The recovery of motor function in SCI rats evaluated by Basso, Beattie and Bresnahan locomotor rating scale (BBB scale); Hematoxylin-eosin staining (scale bar = 50 μm); Nissl staining;. The body weight of the rats was recorded and the animal behavior, including 50% withdrawal threshold and withdrawal latency, was analyzed in the rats. ‘^*^’ indicates *p* < 0.05.

After acute SCI in rats, the loss of neuronal function and microscopical disturbances impair motor function. In this study, the BBB scale was applied to assess the recovery of motor function in SCI rats. The results indicated that the motor function decline occurred in the SCI group and PEA group, but the BBB score remarkably increased in the PEA group on the 7th day (vs. SCI group, *p* < 0.05) (Figure 1A), suggesting that the PPARα agonist promotes SCI motor function recovery. To explore whether the motor function recovery, promoted by the PPARα agonist, is related to neuronal survival, the nerve tissues were sampled on the 7th day of treatment of SCI conditions were observed using H&E staining (Figure 1B) and Nissl staining (Figure 1C). The results showed that the SCI group had obvious destruction of the spinal cord tissue structure and visible tissue edema and inflammatory cells’ infiltration, moreover, the number of neurons was decreased significantly. In contrast, the inflammatory cells in the spinal cord tissues were decreased, the tissue edema was alleviated, the number of neurons was increased, and the structure was relatively complete in the PEA group. Meanwhile, the 50% withdrawal threshold and withdrawal latency were repressed in SCI group, while the treatment of PEA induced the phenotypes in the rats, with no difference in body weight (Figure 1D).

### PPARα agonist relieved inflammatory responses in SCI rats by inhibiting NF-κB expression

**Figure 2:**
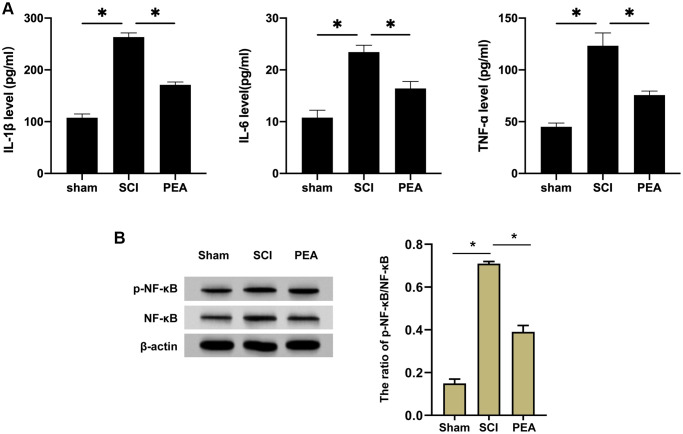
**PPARα agonist relieved inflammatory responses in SCI rats by inhibiting NF-κB expression.** (**A** and **B**) The rats were randomly divided into sham-operation group (Sham group), rat SCI model group (SCI group), SCI + PPARα agonist PEA group (PEA group). The SCI rat model was established using modified Allen's method. The rats in the PEA group were intraperitoneally injected with PEA (2 mg/kg). The recovery of motor function in SCI rats evaluated by BBB scale; The levels of serum inflammatory factors (interleukin-1 beta (IL-1β), interleukin-6 (IL-6) and tumor necrosis factor-alpha (TNF-α)) detected by ELISA; The protein expression and phosphorylation levels of NF-κB detected by Western Blotting in spinal cord tissues; ‘^*^’ indicates *p* < 0.05.

After SCI, inflammatory cells can invade the impaired spinal cord and trigger inflammatory responses, thus aggravating the damage [9, 25, 26]. The rats’ serum inflammatory factors from each group were first detected by ELISA. We observed that the content of IL-1β, IL-6 and TNF-α was notably raised in the SCI group, while it was reduced in the PEA group (vs. SCI group, *p* < 0.05) (Figure 2A). TNF-α pro-inflammatory effect in SCI depends on the activity of NF-κB; therefore, the expression of NF-κB in the spinal cord was analyzed [27]. According to the results, the PPARα agonist could inhibit the expression and the phosphorylation of NF-κB in the SCI model (Figure 2B), implying that the PPARα agonist could relieve inflammatory responses in SCI rats by repressing NF-κB expression and phosphorylation.

### PPARα agonist inhibited oxidative stress injury in SCI rats

**Figure 3:**
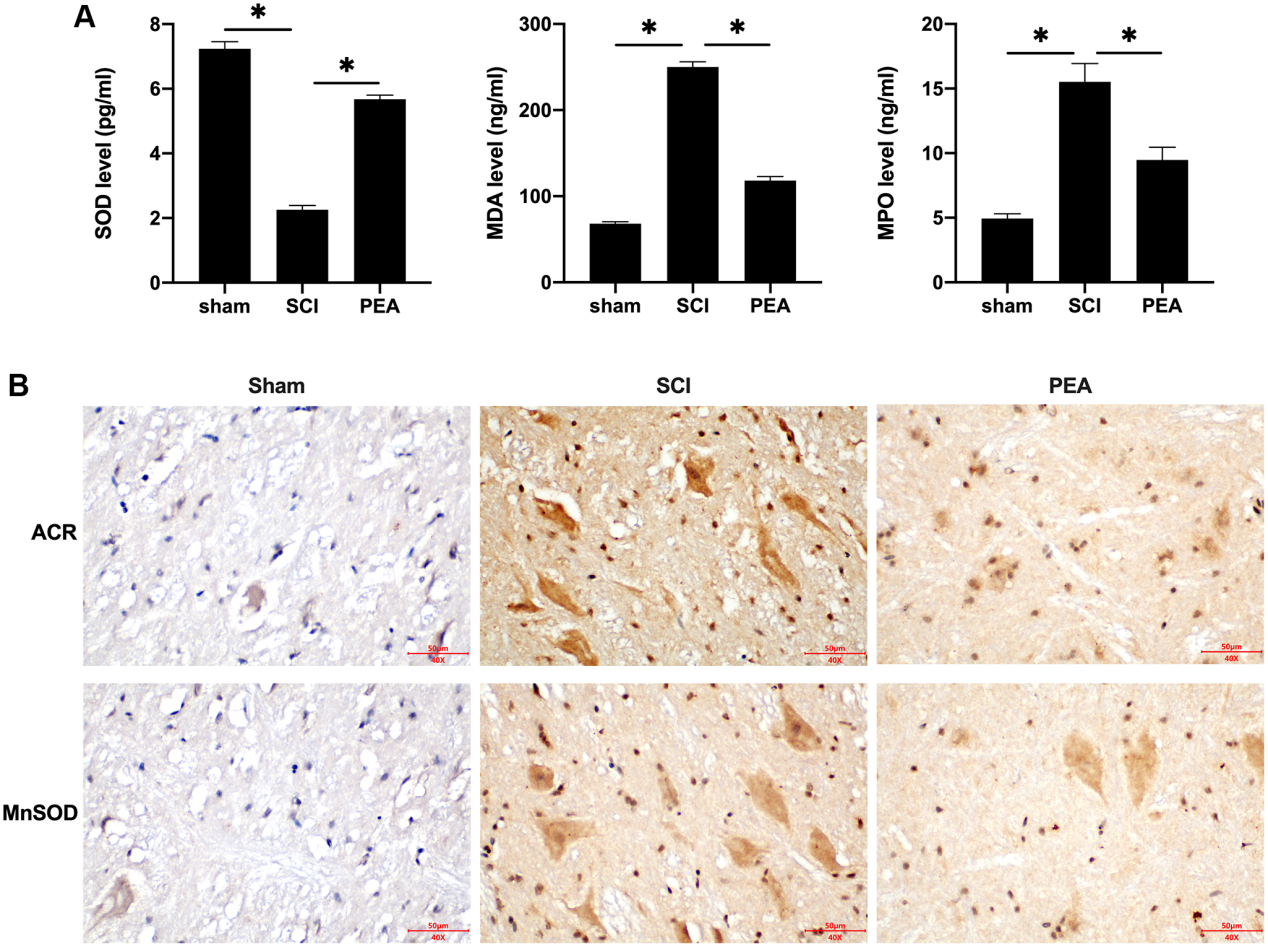
**PPARα agonist inhibited oxidative stress injury in SCI rats.** (**A** and **B**) The rats were randomly divided into sham-operation group (Sham group), rat SCI model group (SCI group), SCI + PPARα agonist PEA group (PEA group). The SCI rat model was established using modified Allen's method. The rats in the PEA group were intraperitoneally injected with PEA (2 mg/kg). Serum content of Oxidative stress factors (superoxide dismutase (SOD), malondialdehyde (MDA) and myeloperoxidase (MPO)) detected by ELISA; The expression of reactive oxygen species (ROS) (acrylamide (ACR) and manganese superoxide dismutase (MnSOD)) detected by IHC in spinal cord tissues; ‘^*^’ indicates *p* < 0.05.

Following SCI, apparent inflammatory changes may emerge in the impaired spinal cord, and the spinal cord tissues, that are abundant in lipids, are particularly sensitive to lipid peroxidation [28]. In this study, the activity of SOD was inhibited, while the content of MDA and myeloperoxidase [29] was evidently increased in the SCI rat model (*p* < 0.05) (Figure 3A). However, the PPARα agonist could enhance SOD activity and suppress MDA and MPO secretions. Furthermore, ACR and MnSOD, the products of oxidative stress with stronger toxicity and longer half-life, were examined. The results revealed that the PPARα agonist could down-regulate ACR expression and up-regulate MnSOD expression in the spinal cord of SCI rats (Figure 3B). These results suggest that the PPARα agonist can inhibit oxidative stress injury in SCI rats.

### PPARα agonist alleviated neuronal apoptosis in spinal cord of SCI rats by activating the PI3K/Akt signaling pathway

**Figure 4:**
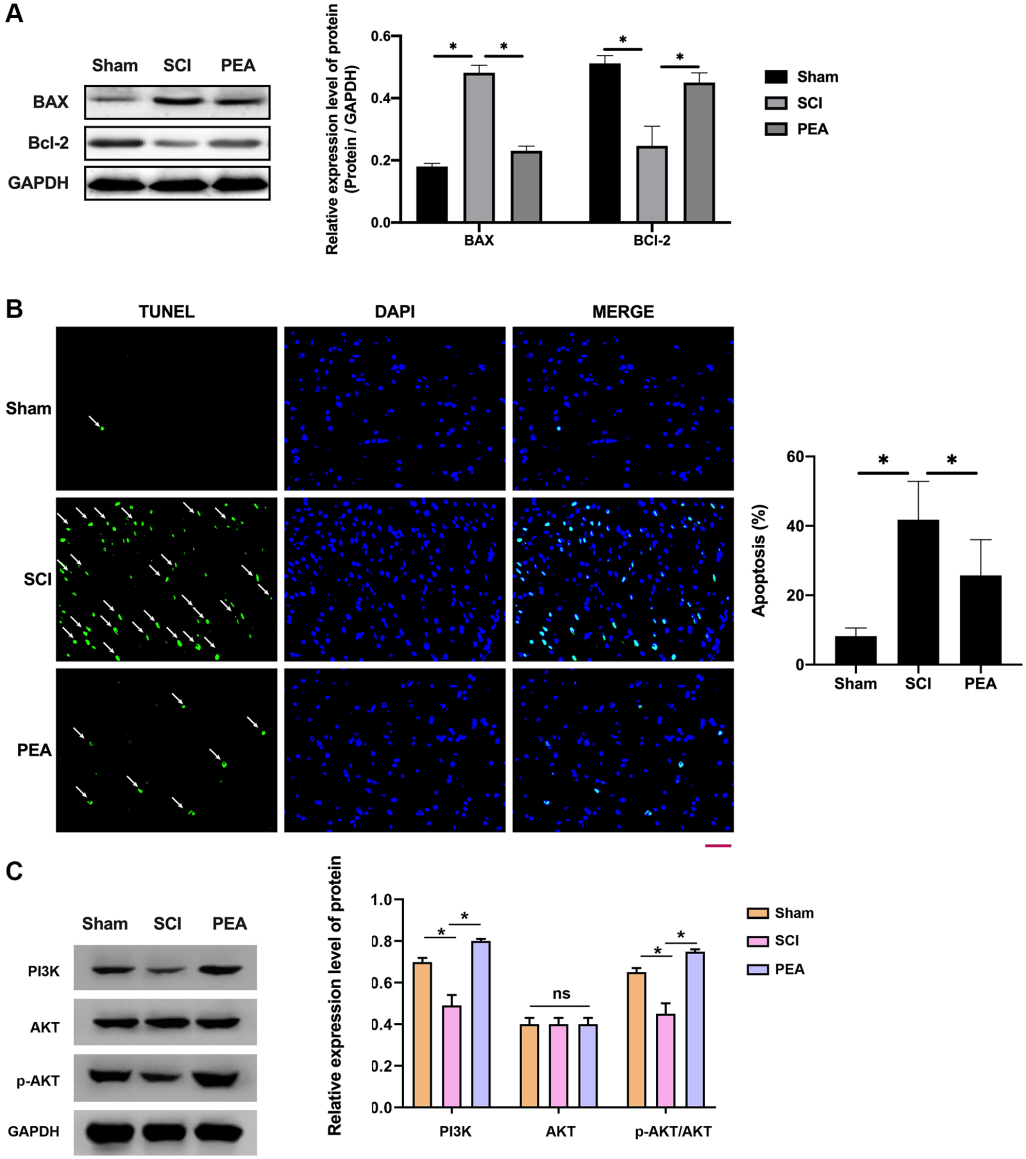
**PPARα agonist alleviated neuronal apoptosis in spinal cord of SCI rats by activating the PI3K/Akt signaling pathway.** (**A**–**C**) The rats were randomly divided into sham-operation group (Sham group), rat SCI model group (SCI group), SCI + PPARα agonist PEA group (PEA group). The SCI rat model was established using modified Allen's method. The rats in the PEA group were intraperitoneally injected with PEA (2 mg/kg). Western Blotting was performed to detect apoptosis factors of Bax and Bcl-2 in spinal cord tissues; The apoptosis rate detected by TUNEL staining (scale bar = 50 μm) in spinal cord tissues; The ImageJ software was used to analyze the TUNEL-positive cells, which represented the apoptosis rate. The expression of PI3K/AKT signaling pathway-related proteins detected by Western Blotting in spinal cord tissues; ‘^*^’ indicates *p* < 0.05.

When SCI occurred, the cells stimulated by oxidative stress could activate the intrinsic apoptosis pathway [30] to elevate the expression level of the apoptotic activator Bax and lower that of the anti-apoptotic factor Bcl-2 in the spinal cord (*p* < 0.05) (Figure 4A); thereby leading to apoptosis. However, the PPARα agonist could reduce the neurons’ apoptosis rate (*p* < 0.05) (Figure 4B), suggesting a potential mechanism of action PI3K activation which could phosphorylate and activate Akt, prompting its action on downstream target proteins of the signaling pathway (*p* < 0.05) (Figure 4C).

### PPARα agonist activated the Nrf2/HO-1 signaling pathway

**Figure 5:**
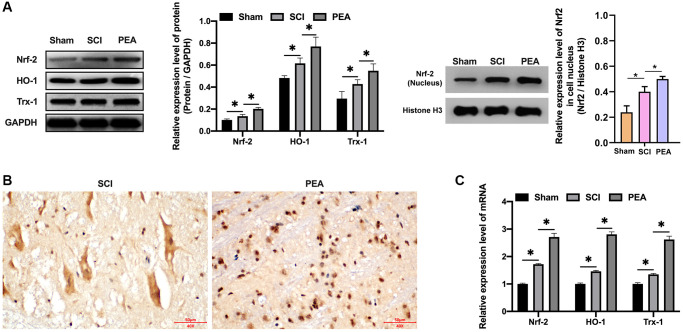
**PPARα agonist activated the Nrf2/HO-1 signaling pathway.** (**A**–**C**) The rats were randomly divided into sham-operation group (Sham group), rat SCI model group (SCI group), SCI + PPARα agonist PEA group (PEA group). The SCI rat model was established using modified Allen's method. The rats in the PEA group were intraperitoneally injected with PEA (2 mg/kg). The expression of Nrf2/HO-1 signaling pathway-related proteins detected by Western Blotting in spinal cord tissues; The expression of Nrf2 detected by IHC in spinal cord tissues; Gene transcription and expression of anti-oxidative protein (HO-1 and Trx-1) detected by qRT-PCR in spinal cord tissues; ‘^*^’ indicates *p* < 0.05.

As the body was in an oxidative stress state when SCI occurred, Nrf2 was activated [31], the degradation rate simultaneously declined and the nuclear content of Nrf2 was markedly raised, accordingly, the protein expression levels of HO-1 and Trx-1 were both drastically decreased (*p* < 0.05) (Figure 5A). In contrast, the PPARα agonist could stimulate Nrf2 entering the nucleus (Figure 5B and Supplementary Figure 1A), its binding to antioxidant response elements (AREs); therefore, initiating the transcription and expression of downstream anti-oxidant proteins, such as HO-1 and TRX-1 of AREs (*p* < 0.05) (Figure 5C), thus providing a defense mechanism against internal and external harmful stimuli.

### PPARα agonist promoted motor function repair in SCI rats by activating the Raf-1/MEK/ERK signaling pathway

**Figure 6:**
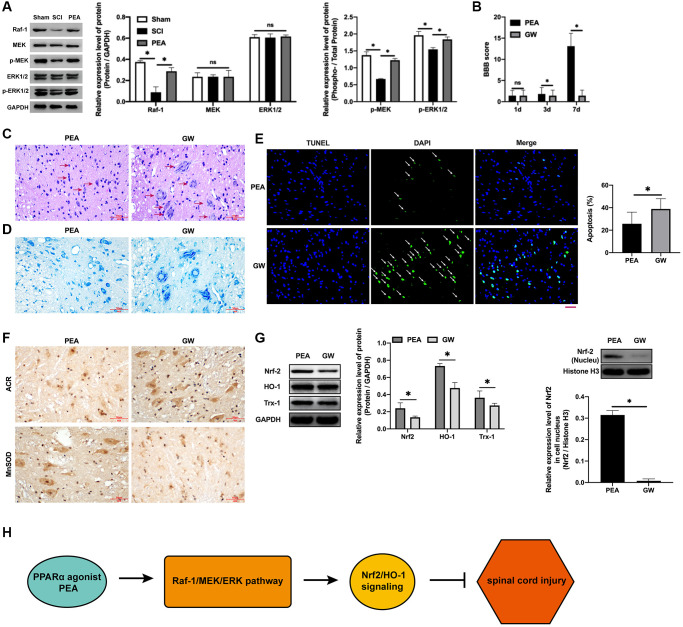
**PPARα agonist promoted motor function repair in SCI rats by activating the Raf-1/MEK/ERK signaling pathway**. (**A**–**G**) The rats were randomly divided into sham-operation group (Sham group), rat SCI model group (SCI group), SCI + PPARα agonist PEA group (PEA group) and SCI + PEA + Raf-1 inhibitor GW5074 group (GW group). The rats in the PEA group were intraperitoneally injected with PEA (2 mg/kg). The GW group were administered with Raf-1 inhibitor GW5074 (5 mg/kg) based on the PEA group treatment. The expression of the V-raf-1 murine leukemia viral oncogene homolog 1 (Raf-1)/mitogen-activated protein kinase (MEK)/extracellular signal-regulated kinase (ERK) signaling pathway-related protein detected by Western Blotting in spinal cord tissues; The BBB score; HE staining (scale bar = 50 μm); The number of neurons; The expression of ROS (ACR and MnSOD) detected by IHC in spinal cord tissues; The expression of apoptosis factors (Bax and Bcl-2) detected by Western Blotting in spinal cord tissues; Protein expression levels of nuclear factor erythroid 2-related factor 2 (Nrf2), heme oxygenase 1 (HO-1) and thioredoxin-1 (Trx-1) detected by Western Blotting in spinal cord tissues; (**H**) A diagram model of this study. ‘^*^’ indicates *p* < 0.05.

In the case of SCI, the Raf-1/MEK/ERK signaling pathway transmitted the extracellular stimulation signals into the nucleus, which regulate the expression of stress proteins and ultimately achieving the response to extracellular stimuli [11, 24]. The PPARα agonist could facilitate the phosphorylation of MEK and ERK (*p* < 0.05) (Figure 6A). Nevertheless, after the administration of the Raf-1 inhibitor, the BBB score decreased (*p* < 0.05) (Figure 6B), SCI worsened (Figure 6C), the number of neurons reduces (Figure 6D), increased apoptotic rate (*p* < 0.05) (Figure 6E), ACR expression elevated and MnSOD expression inhibited (Figure 6F), protein expression levels of Nrf2, HO-1 and Trx-1 decreased (*p* < 0.05) (Figure 6G and Supplementary Figure 1B), and the protection of the PPARα agonist SCI attenuated, implying that the PPARα agonist can promote SCI rats’ motor function repair by activating the Raf-1/MEK/ERK signaling pathway.

## DISCUSSION

Soon after SCI, the infiltration of bone marrow-derived neutrophils occurs at the injury site where they release cytokines and lytic enzymes, resulting in tissue damage exacerbation and oriented chemotaxis of other inflammatory cells. PPARα exerts its anti-inflammatory effects by transcriptionally regulating gene expression of relevant inflammatory factors [11]. In the present study, the established SCI models were treated with a PPARα agonist and we found that it could improve SCI rats’ motor function, mitigate SCI and inhibit inflammatory responses and oxidative stress injury. The associated mechanism may involve the Raf-1/MEK/ERK and Nrf2/HO-1 signaling pathways.

PPARα performs its biological functions through several pathways, is mainly involved in the transcriptional regulation of target genes via the ligand-dependent transcriptional activation. If there is a lack of specific ligands, PPARα will recruit several co-inhibitory factors to inactivate target genes, a process known as ligand-independent transcriptional repression [32, 33]. Crisafulli et al. used LPS to induce peritoneal macrophages in wild-type and PPARα-/- rats and the results showed that the expression of multiple inflammatory indexes was distinctly higher in the peritoneal macrophages of PPARα-/- rats than that of wild-type rats. PPARα regulates the anti-Inflammatory impact of melatonin in SCI [13]. The knockout mice present an induced inflammatory activity in SCI [11]. However, other serious disease phenotypes in the SCI model constructed in mice knocked out of the PPARα gene should be explored in future investigations. Meanwhile, PEA has been reported to play crucial roles in multiple diseases. It has been reported that PEA represses colon inflammation by the activation of PPARα in an enteric glia/toll like receptor 4-dependent manner [34]. PEA regulates retinal fibrosis and neovascularization by affecting PPARα [35]. PEA modulates hepatic metabolic inflexibility by affecting mitochondrial efficiency and function in diet-stimulated obese mice [36]. Moreover, it has been found that PPAR-γ and PPAR-δ are involved in neuroprotective activities and anti-inflammatory effect of PEA in SCI [29]. The co-ultramicronized treatment of luteolin and PEA prevent neuroinflammation in SCI [37]. The treatment of PEA relieves central neuropathic pain in spinal cord injury [38]. The co-ultramicronized treatment of luteolin and PEA contributes to the neuronal regeneration in SCI [39]. According to the results of the study, the PPARα agonist could inhibit the secretion of IL-1β, IL-6 and TNF-α and repress NF-κB expression in SCI rats, suggesting that the PPARα agonist relieves SCI in rats by suppressing inflammatory responses.

Free radical-mediated oxidative stress response is regarded as one of the key factors for secondary SCI. The injury mechanism involves ACR induction of lipid peroxidation and the stimulation of oxidative stress response into a vicious cycle, thus causing permanent cell injury. Secondly, ACR can couple with proteins to trigger cell apoptosis [28, 40]. In the guinea pig model of compression-induced SCI *in vitro*, ACR expression was increased at 4 h after SCI and reached a peak at 24 h, which could maintain for 1 week or longer, resulting in persistent ACR damage to the spinal cord. However, the damage to neuronal membranes was significantly alleviated after the administration of specific scavengers. In the present study, SCI rats exhibited obvious oxidative stress and notable ACR increase, which suggested that the induced oxidative stress injury plays important role in the occurrence of motor paralysis and neuropathic pain in rats. Previous studies have demonstrated that Nrf2 can bind to various AREs and mediate the expression of antioxidant proteins and enzymes. Under physiological conditions, Nrf2 combines with the adapter protein Keap1 in the cytoplasm and restrains Nrf2 activity. However, in the oxidative stress state, Nrf2 is dissociated from Keap1, becomes activated and translocate into the nucleus. This event leads to the transcriptional activation of target genes, the regulation of downstream antioxidant proteins synthesis and the enhancement of cell resistance to oxidative stress. In this study, we observed that the PPARα agonist could stimulate Nrf2 translocation into the nucleus, its binding to AREs and the transcriptional initiation of AREs downstream antioxidant proteins (HO-1 and TRX-1); thus, mitigating oxidative stress injury in SCI rats.

In the secondary SCI, massive necrosis or death of neuronal and neuroglia cells occur in tissues. Therefore, inhibiting secondary cell apoptosis, protecting residual neurons and repairing the injured spinal cord and as much as possible, are the directions of current studies on SCI. The Raf/Ras/ERK signaling pathway can transduce the extracellular stimulation signals into the nucleus, regulate the expression of stress proteins and finally accomplish the response to extracellular stimuli.

When ERK is activated, p-ERK can activate the anti-apoptotic factor Bc1-2 which provides resistance to neuronal apoptosis. It has been confirmed that ERK is involved in the regulation of genes related to SCI repair, including RTKs, PTEN and SHANK3. The activation of the ERK signaling pathway is associated with spinal neurodegeneration and dysfunction, caused by SCI in rats. Besides, the ERK inhibitor can prominently relieve secondary SCI and improve rats’ neurological function. In this study, p-ERK was slightly increased after SCI, while the PPARα agonist could facilitate MEK and ERK phosphorylation and remarkably repress neuronal apoptosis, further accelerating neuronal repair and regeneration. Meanwhile, despite we identified that the PPARα agonist PEA could attenuate PEA by regulating the Nrf2/HO-1 and Raf/Ras/ERK signaling pathways, the direct or indirect effect of PPARα on Nrf2/HO-1 and Raf/Ras/ERK signaling remain unclear and should be explored by more investigations.

In conclusion, the PPARα agonist can alleviate SCI in rats, inhibit inflammatory responses, mitigate oxidative stress injury and reduce apoptosis rate and the mechanism correlated with the activation of the Nrf2/HO-1 and Raf/Ras/ERK signaling pathways (Figure 6H).

## MATERIALS AND METHODS

### Laboratory animals and grouping

A total of 40 SPF-grade male Sprague-Dawley rats, weighing 220–260 g, were purchased from Vital River (Beijing, China) and randomly divided into sham-operation group (Sham group), rat SCI model group (SCI group), SCI + PPARα agonist palmitoylethanolamide (PEA) group (PEA group) and SCI + PEA + Raf-1 inhibitor GW5074 group (GW group). This experiment was approved by the Laboratory Animal Ethics and Ethics Committee of General Hospital of Northern Command (Approval No. 2020-03-17).

### Rat model of SCI

The SCI rat model was established using modified Allen's method [41, 42]. The rats were anesthetized by intraperitoneal injection of 2% pentobarbital sodium (3 mL/kg) and fixed on an operation table in the prone position. An incision (2 cm) was made on the posterior midline of the spine to expose the T11 thoracic spinal cord, which was injured by a spinal cord impactor of 50 gcf impact energy (gram × cm × force, impact weight: 5 g, impact height: 10 cm). The successful impact criteria are as follows: spinal hemorrhage and edema at the of injury site, rat tail spasmodic swing, and paralysis of the lower limbs after retraction and fluttering of lower limbs and body. Every day after modeling, the rats in the PEA group were intraperitoneally injected with PEA (Sigma, USA, 2 mg/kg), while those in the GW group were administered with Raf-1 inhibitor GW5074 (Sigma, USA, 5 mg/kg) based on the PEA group treatment. The body weight of the rats was recorded and the animal behavior, including 50% withdrawal threshold and withdrawal latency, was analyzed in the rats.

### Basso, Beattie and Bresnahan locomotor rating scale (BBB scale)

The BBB scale was used to evaluate hindlimbs motor function of in each group of rats at 1, 3 and 7 d after modeling. The rats were placed on a test platform to observe the joint motion of the lower limbs, gait, trunk stability, paws fine motor, tail position and physical coordination. The score ranged from 0 point (complete paralysis without visible hindlimb movements) to 21 points (persistent palm movement, coordinated gait, grounding toes, stable trunk, rising tail, active paw position always parallel to the body during activity and completely normal activity).

### Sample collection and detection

At 7 d after modeling, the rats in each group were anesthetized by pentobarbital sodium and sacrificed by abdominal aorta exsanguinations. The serum was separated by centrifugation and stored at –80°C for later use. Subsequently, injured spinal cord tissues were taken, a part of which was fixed in 10% neutral formalin, and the other part preserved in liquid nitrogen for later use.

### Hematoxylin-eosin staining

The formalin-fixed tissues were gradually dehydrated in 70%, 80%, 90%, 95% and 100% ethanol, transparentized in xylene and paraffin-embedded. Later, the tissue blocks were sliced to 4 μm-thick sections and subjected to H&E staining [43]. Finally, the sections were mounted in neutral balsam and photographed under a light microscope (CX33, Olympus Corporation, Japan).

### Nissl staining

Diparaffinise sections in xylene I and II for 10 minutes each, and hydrate in absolute ethanol I and II for 3 minutes each, followed by 90%, 80% and 70% ethanol for 3 minutes, respectively. Then the slices were rinsed in tap water and in distilled water. After that, stain the slices in 0.1% cresyl violet solution for 10 minutes. Straight afterwards, rinse the slices in distilled water quickly for twice, and then differentiate the slices in 70% and 90% ethanol for 1 minute each. Following this, absolute ethanol II and I for 1 minute each were performed on for dehydration, and slices were cleared with xylene II and I for 1 minute individually. Finally, the slices were permanently mounted with resinene for further microscopically observation and image recordings.

### Immunohistochemical staining

The paraffin-embedded blocks were sliced to sections (4 μm-thick), placed in a 30% hydrogen peroxide solution for 5 min, soaked in 0.01 mol/L citric acid buffer solution (pH = 6.0) after washing in water and heated in a microwave oven for antigen retrieval. Then, the sections were put into a wet box, drops of 5% BSA blocking buffer were added and placed at room temperature for 20 min. After excess liquid removal, acrylamide (ACR) (Abcam, USA) and manganese superoxide dismutase (MnSOD) (Abcam, USA) antibodies were diluted at 1:500 and added dropwise and the sections (in the wet box) placed in a refrigerator at 4°C overnight, washed 3 times with phosphate-buffered saline (PBS), added with drops of secondary antibodies, incubated at 37°C for 30 min, washed again 3 times with PBS and color developed using a DAB kit. Finally, the sections were mounted in neutral balsam and photographed under the light microscope.

### Terminal deoxynucleotidyl transferase-mediated dUTP nick end labeling (TUNEL) staining

Spinal cord neuronal apoptosis was determined using a TUNEL staining kit (C1086, Beyotime, China). The paraffin-embedded sections were twice deparaffinized in xylene (5 min/time), immersed in gradient alcohol and added dropwise with DNase-free (20 μg/mL) proteinase K (20 mg/mL). After 30 min of incubation at 37°C, the sections were twice flushed with PBS, added with 50 μL of TUNEL staining solution and incubated in the dark at 37°C for 60 min, washed for 3 times in PBS, drops of DAPI staining solution were added and incubated in the dark at room temperature for 10 min. After another 3 times washing step in PBS, the sections were mounted in anti-fade fluorescence mounting medium and photographed under a fluorescence microscope.

### Enzyme-linked immunosorbent assay (ELISA)

The serum content in IL-1β, IL-6, tumor necrosis factor-alpha (TNF-α), malondialdehyde (MDA), superoxide dismutase (SOD), and MPO in each group of rats was measured using ELISA kits (Abcam, USA) and in accordance with the manufacturer’ instructions. In brief, standard substances and test samples were sequentially added into ELISA plates and incubated at 37°C for 60 min. The plates were washed in a plate washer, added with 50 μL of ELISA reagent and incubated at 37°C for 30 min. After washing, 50 μL of color developer A and 50 μL of color developer B were separately added for color development in the dark at 37°C for 15 min. Following this step, 50 μL of stop buffer was added into each well, and the absorbance (at 450 nm) was determined using a microplate reader. Finally, the content of samples was calculated based on the standard curves.

### Western blotting

The spinal cord tissues were taken out from liquid nitrogen, lysed in RIPA lysis buffer, containing protease inhibitor, for 30 min and centrifuged to obtain the supernatant which was used to determine the protein concentration. After SDS-PAGE, the proteins were transferred onto a membrane, sealed and incubated overnight at 4°C with antibodies against NF-κB (Abcam, USA), p-NF-κB (Abcam, USA), B-cell lymphoma-2 (Bcl-2) (Abcam, USA), Bcl-2-associated X protein (Bax) (Abcam, USA), phosphatidylinositol 3-kinase (PI3K) (Abcam, USA), protein kinase B (Akt) (Abcam, USA), phosphorylated (p)-Akt (Abcam, USA), heme oxygenase-1 (HO-1) (Abcam, USA), Nrf2 (Abcam, USA), trithorax-1 (TRX-1) (Abcam, USA), Raf-1 (Abcam, USA), MEK (Abcam, USA), ERK (Abcam, USA), p-MEK (Abcam, USA), p-ERK (Abcam, USA) and glyceraldehyde-3-phosphate dehydrogenase (GAPDH) (Abcam, USA) (diluted at 1:1000). Next, the PVDF membrane (Sigma, USA) was cleansed in TBST and incubated with horseradish peroxidase-labeled secondary antibodies at room temperature for 2 h. Finally, the color of the proteins was developed using an ECL kit and gel imaging system, and the absorbance analyzed by Image Tools.

### Quantitative reverse transcription-polymerase chain reaction (qRT-PCR)

The liquid nitrogen stored spinal cord tissues were added with 1 mL of TRIzol (15596026, Invitrogen, USA) and RNA was extracted via the TRIzol-chloroform method and reversely transcribed into cDNA (K1622, Invitrogen, USA). Subsequently, primers of HO-1, Nrf2, PKC-a and GAPDH were added separately, and a reaction system was prepared using a qRT-PCR kit (204057, Qiagen, USA) and put in a PCR instrument. The primers’ sequences were listed in Table 1. The reaction conditions for 40 cycles in total were as follows: pre-denaturation at 95°C for 30 s, and PCR at 95°C for 5 s and at 60°C for 20 s. The results were presented as 2^−ΔΔCt^.

**Table 1:** Primer sequences.

**GENE NAME**		**GENE PRIMER**
HO-1	Forward:	AGTAGGCCACATTACACTGCT
	Reverse:	GACCCACACCTCACAAATTGA
Nrf2	Forward:	TCATGTGGTCAAGACGAGAT
	Reverse:	GGAGGAAGGAAGGCATGA
TRX-1	Forward:	AGAATGAGGACTGGGTGAGAAAC
	Reverse:	CAACGGCTCTGGATAAAGTGTCTA
GAPDH	Forward:	GCACACAGTACATCCGTCA
	Reverse:	TTCTCCGAACGTGTCACGT

### Statistical analysis

The SPSS 19.0 software was used for statistical analysis of the experimental results and the data were expressed as mean ± standard deviation. Statistical significances between two groups or multiple groups were determined by Student’s *t*-test or one-way ANOVA. Moreover, corrected *t*-test was used for comparing the experimental data with variance homogeneity between two groups. *P* < 0.05 suggested that the difference between two experimental groups was statistically significant.

## Supplementary Materials

Supplementary Figure
