# Supplementary material for: PPARα agonist relieves spinal cord injury in rats by activating Nrf2/HO-1 via the Raf-1/MEK/ERK pathway
Source: Aging (Albany NY). 2021 Nov 19;13(22):24640–54. doi: 10.18632/aging.203699 (PMC8660597; doi:10.18632/aging.203699)
Supplement: Supplementary Figure [file aging-13-203699-s001.pdf]

SUPPLEMENTARY FIGURE

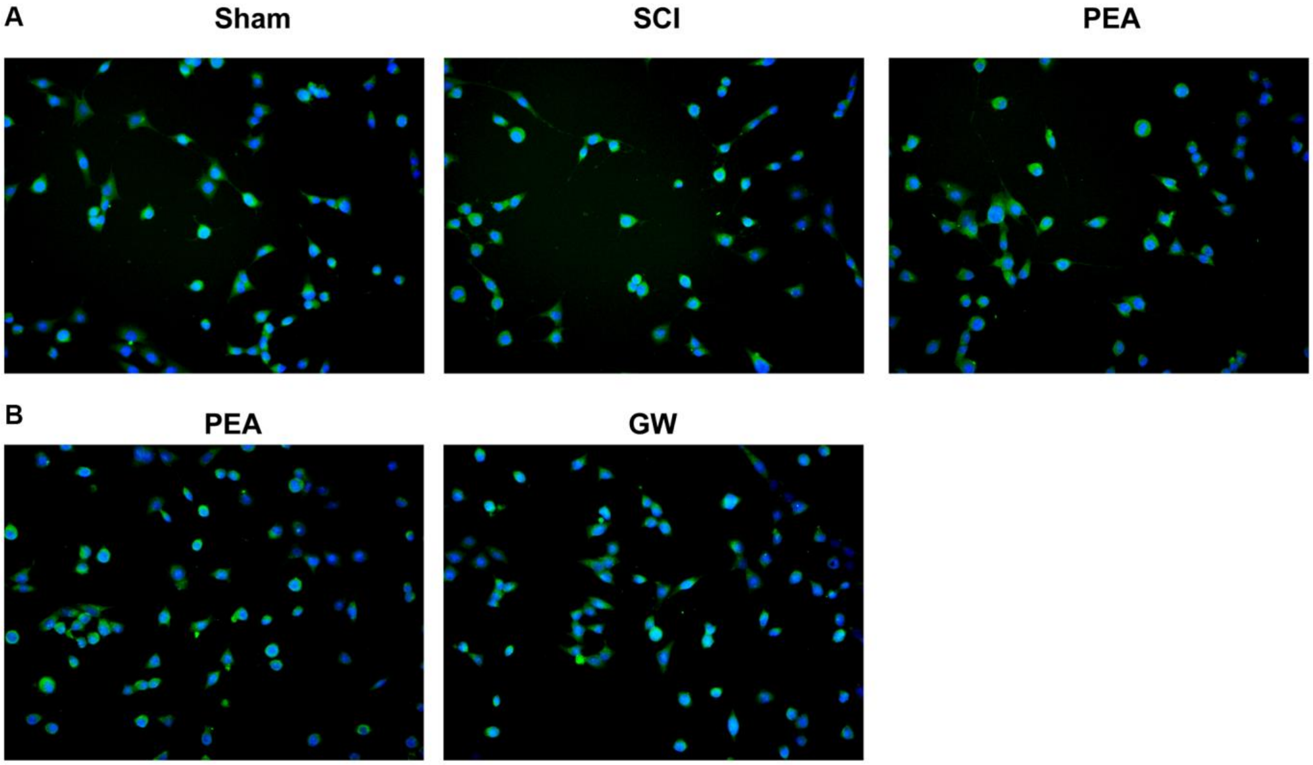

Supplementary Figure 1. The immunofluorescence analysis of Nrf2. (**A** and **B**) The rats were randomly divided into sham-operation group (Sham group), rat SCI model group (SCI group), SCI + PPARα agonist PEA group (PEA group) and SCI + PEA + Raf-1 inhibitor GW5074 group (GW group). The immunofluorescence analysis of Nrf2 was shown.
